# Mitochondrial DNA induces Foley catheter related bladder inflammation via Toll-like receptor 9 activation

**DOI:** 10.1038/s41598-018-24818-w

**Published:** 2018-04-23

**Authors:** Carlos A. Puyo, Alexander Earhart, Nicholas Staten, Yuan Huang, Alana Desai, Henry Lai, Ramakrishna Venkatesh

**Affiliations:** 1 Department of Anesthesiology and Critical Care, Washington University School of Medicine in St. Louis, St. Louis, Missouri USA; 2 Department of Surgery, Washington University School of Medicine in St. Louis, St. Louis, Missouri USA

## Abstract

Bladder instrumentation engages the innate immune system via neutrophil activation, promoting inflammation and pain. Elevated levels of mitochondrial DNA (mtDNA) have been associated with tissue damage and organ dysfunction. We hypothesized that local bladder trauma induced by a Foley catheter (FC) will result in mtDNA release, migration of neutrophils into the bladder lumen, and activation of the Toll-like receptor 9 (TLR9) and nuclear factor kappa B (NF-κB) pathway leading to bladder tissue damage. We randomized 10 swine into two groups receiving uncoated, or chloroquine/N-Acetylcysteine (CQ/NAC)-coated FCs. Urine samples were analyzed for mtDNA activation of TLR9/NF-κB as demonstrated by indicators of neutrophil adhesion, migration, and activation. We found that uncoated FCs resulted in a unique active neutrophil phenotype that correlated with bladder epithelial injury, neutrophilia, necrosis, mtDNA release, TLR9/NF-κB activation, transcription and secretion of pro-inflammatory cytokines, and enhanced respiratory burst. In our study we observed that the high levels of mtDNA and elevated TLR9/NF-κB activity were ameliorated in the CQ/NAC-coated FC group. These findings suggest that post-migrated bladder luminal neutrophils are involved in local tissue damage and amelioration of the mtDNA/TLR9/NF-κB inflammatory axis may represent a therapeutic target to prevent inflammation, and bladder tissue injury.

## Introduction

Bladder catheterization is common during hospitalization and may acutely induce tissue damage evident by bladder irritation (cystitis), spasms, pain and urinary infections^1,2^, all possible causes of delayed hospital discharge and increased medical costs. Although many studies have shown the impact of bladder uropathogens on local and systemic inflammation^3,4^, the primary factors involved in early neutrophil activation in the absence of bacterial contamination are not completely elucidated and require a better understanding of the cellular and molecular mechanisms present during a sterile injury. We have previously shown that human and swine mucosal invasion by a foreign body induced mucosal inflammation and activation of the innate immune system resulting in neutrophil infiltration^5,6^. It is known that bladder instrumentation with a Foley catheter (FC) results in inflammation and neutrophil cell recruitment, thus promoting bacterial contamination^7,8^. Neutrophil cells are essential for a proper innate immune response as they have the capacity to express multitude of surface and intracellular receptors to contain and destroy injurious sterile products^9–11^. For instance, as neutrophils migrated through tissues they will express surface receptors indicative of epithelial/neutrophil interaction as seen by expression of ICAM-1 (CD54)^12,13^. Neutrophils also have the capacity to release various pro-inflammatory cytokines in order to promote cellular responses, for instance tumor necrosis factor (TNF-α), interleukins (IL)-1β and IL-6, may impact several cellular activities ranging from delayed apoptosis to tissue and cell necrosis^9,14–17^. Less is known about local neutrophil activity mediated by non-bacterial triggers of neutrophil activation in bladder injury.

Neutrophils are first responders of the innate immune system against infectious and non–infectious agents following detection by pattern-recognition receptors (PRRs), which include the family of Toll-like receptors (TLRs)^18,19^. TLRs identify not only bacterial antigens but also host sterile intracellular molecules (damage associated molecular patterns, DAMPs)^20–22^ released during cellular injury, generating signals that induce inflammatory activity. The urinary system express several TLRs, such as TLR4, TLR5 and TLR11 on cells lining the urinary tract, were they contribute to the local immune defense^18,23,24^. TLRs and in particular TLR9 can be activated by evolutionarily conserved pathogen–associated molecular patterns (PAMPs) such as unmethylated CpG DNA present in bacteria, or by oxidized mtDNA of non-bacterial origin^25,26^. More recent work has demonstrated that oxidized mitochondrial DNA (mtDNA) released during eukaryotic cell injury induce neutrophil expression of TLR9, thus mediating neutrophil activation^27^. In particular, the effects of mtDNA/TLR9 signaling has been documented under sterile conditions, indicating that mtDNA is a TLR9 agonist that can activate downstream pro-inflammatory pathways such as nuclear factor kappa B (NF-κB)^28–30^. TLR9 is known to signal via MyD88-dependent pathway, which ultimately activates NF-κB gene transcription of as many as 400 genes with heterogeneous actions ranging from apoptosis, to inflammation and cell activity regulation^28^. The importance of mtDNA on organ dysfunction has been illustrated in multiple reports linking sterile inflammation to cardiac dysfunction^31^, arthritis^32^, tracheal injury^33^ and other pathologies^34,35^. Indeed, recently we showed that mtDNA induced TLR9/NF-κB mediated neutrophil activation that resulted in tracheal mucosal inflammation and pain (sore throat) in human subjects exposed to an endotracheal tube^33^. Neutrophil migration during bladder infection has been shown to correlate with bacterial load^36,37^, however, the role of necrotic cell products such as mtDNA on TLR9 and neutrophil activation during presence of a Foley catheter (FC) has not been elucidated.

In this study, we hypothesized that a sterile bladder injury induced by FC placement can initiate a local inflammatory response that promotes neutrophil migration predominantly dependent on TLR9 activation. Here we show in a swine model that FC placement results in loss of bladder mucosa integrity with subsequent neutrophil cell migration and activation mediated by necrosis, resulting in local mtDNA release, and TLR9/NF-κB activation. We aimed to understand the impact of sterile injury induced by a Foley catheter by analyzing the effects of disrupting intralysosomal activation^38,39^ of TLR9 with chloroquine (CQ), and N-Acetylcysteine (NAC)^40,41^ which can downregulate the pro-inflammatory action of NF-κB and thus decrease cytokine activity.

## Materials and Methods

### Study Design

Five size 10 French Dover™ FCs were dipped in a CQ/NAC mixture (20% PVP/5% Mg-Stearate/35 µM CQ/10 mM NAC in 10% ethanol) for 15 minutes, then dried in a sterile incubator at 37 °C + 5% CO_2_ for 15 minutes, and repeated twice. The coated tubes were then dried overnight and resealed under sterile conditions.

After approval by the Animal Studies Committee at Washington University Medical School in St. Louis, and in accordance to the guidelines stipulated by the Animal Welfare Act and the Association for the Assessment and Accreditation of Laboratory Animal Care (AAALAC), we conducted a prospective study to evaluate the impact on bladder tissue and neutrophil activation for 6 hours of exposure to uncoated and CQ/NAC-coated FCs in a swine model. Ten female swine were randomized to the two groups (5 uncoated, 5 CQ/NAC-coated), and anesthetized with 1 to 2 mg/kg tiletamine, ketamine, xylazine mix and maintained with 1–3% isoflurane followed by catheterization. FC balloons were inflated with 10 mL saline. Approximately 20 mL urine samples were drawn after catheterization, and each hour afterwards, with 5 mL blood drawn simultaneously from a peripheral vein. After FC removal, tissue samples of the bladder and urethra at point of contact were taken, immediately after the swine were euthanized.

### Extracellular Marker Quantitation

Twenty milliliters of urine from each time point was centrifuged to separate cells and extracellular components. Mitochondrial DNA (mtDNA) concentrations within the urine supernatants were assessed by real time quantitative polymerase chain reaction (RT-qPCR) for swine mitochondrial-encoded cytochrome b (*Mt-cyb*), and compared against a standard curve of known mtDNA concentrations (Bio-Rad Laboratories Inc, Hercules, CA, USA). Urine supernatant samples were adjusted to between 6.5–7.5 pH with 10 N NaOH.

Concentrations of swine inflammatory markers IL-1β, IL-6, IL-8, IL-10, and TNF-α in 25 μL of urine were measured by LuminexTM xMAP technology using porcine ProcartaPlexTM Simplex Kits (Life Technologies Inc, Carlsbad, CA, USA) against a standard curve of known concentrations for each marker. The amount of NaOH was negligible and thus not factored into the dilution. Activity of secreted neutrophil elastase (NE) in urine supernatants was measured by hydrolysis of the chromogenic substrate N-methoxysuccinyl-Ala-Ala-Pro-Val p-nitroanilide (Sigma Aldrich Inc, St. Louis, MO, US) into 4-nitroaniline after incubating for 1 hour at 37 °C + 5% CO_2_ by light absorbance at 405 nm via spectrophotometry with a NanoDrop 2000 (Thermo Fisher Scientific).

### Neutrophil Characteristics

Neutrophils from the urine cell pellets were isolated by magnetic negative separation using EasySep™ Neutrophil Enrichment Kits (Stem Cell Technologies) per manufacturer’s instructions. Half of the neutrophils were stained with antibodies for CD11a (clone BF2L1), CD11b (clone ICRF44), CD16 (clone G7), CD18 (clone TS1/18), CD54 (clone 15.2), and CD62L (clone SK11) as activity and adhesion markers, and 7-Aminoactinomycin D and Annexin V as viability markers, while neutrophil respiratory burst was characterized by incubating cells for 10 mins at 37 °C with 10 ng/mL phorbol 12-myristate 13-acetate followed by 10 seconds with 20 μM dihydrorhodamine 123 (DHR123) and immediately characterized by flow cytometry (FACScan DxP10 flow cytometer, BD Biosciences, San Jose, CA, USA). Ten thousand events were analyzed with FlowJo® X software (FlowJo, LLC, Tree Star, Ashland, OR, USA). The remaining urine neutrophils were saved for mRNA extraction with TRIzol® (Thermo Fisher Scientific) per manufacturer’s instructions, and transcription of *Il1β*, *Il6*, *Il8*, *Il10*, and *Tnfα* (primers from Thermo Fisher Scientific) was evaluated compared against mRNA from freshly isolated peripheral blood neutrophils via RT-qPCR and run with the Bio-Rad CFX 96 system and analyzed with the Bio-Rad CFX Manager software (Bio-Rad Laboratories Inc).

### TLR9 Cell Reporter Assay

A human embryonic kidney 293 (HEK 293) cell line containing TLR9 and NF-κB/secreted embryonic alkaline phosphatase (SEAP) genes (HEK-Blue™ hTLR9; Invivogen, San Diego, CA, USA) was grown out according to manufacturer instructions at 37 °C + 5% CO_2,_ passaging cells every three days. After three passages, approximately 8 × 10^4^ cells in 100 μL HEK 293 growth media (DMEM with 4.5 g glucose, 2 mM glutamine, 10% fetal bovine serum, 20 U/mL penicillin/streptomycin, 100 μg/mL NormocinTM/ZeocinTM antibiotics) were treated with 100 µL urine from coated and uncoated swine groups and incubated at 37 °C + 5% CO_2_ for 4 hours with no treatment, with 35 µM CQ or 10 mM NAC, or combined 35 µM CQ/10 mM NAC; and also 10 μM oligodeoxynucleotide (ODN, or ODN 2006) and inhibitory ODN (iODN, or ODN TTAGGG A151, InvivoGen) as positive and negative controls, respectively. Activation of NF-κB via TLR9 in these cells was determined by calorimetric detection of SEAP release, as measured by hydrolysis of 20 μL QUANTI-Blue™ detection medium (Invivogen) after incubation for 1 hour at 37 °C + 5% CO_2_ via the Synergy H1 micro-plate reader (BioTek Instruments Inc, Winooski, VT, USA) at 655 nm light absorbance.

### Histology and immunohistochemical staining for TLR9

Biopsy samples of urethra and bladder neck were fixed in 10% formalin between 48–72 hours, washed and dehydrated in decreasing ethanol concentrations at 100%, 70%, 50%, and 30%, then paraffin-embedded, and stained with hematoxylin and eosin (H&E). A veterinary pathologist blinded to the samples scored local epithelial tissue injury/integrity, and inflammation as established by numerical scoring guidelines from 0–4, with a score of 0 representing normal to minimal injury and no infiltrating immune cells, and 4 representing extensive diffuse injury and marked immune cell infiltration^42^.

Paraffin-embedded samples from both groups were deparaffinized by washing in xylene, and then washed in decreasing ethanol concentrations at 100%, 95%, 70%, and 50%, followed by rinsing with cold water. Samples were then covered in a Tris-EDTA antigen retrieval buffer (10 mM Tris base, 1 mM EDTA, 0.05% Tween-20, at pH 9.0) and heated to 98 °C in a pressure cooker for 3 minutes, followed by cooling for 10 minutes. Slides were then washed twice with TBS + 0.025% Triton X-100 for 5 minutes, followed by blocking in 10% FBS + 1% BSA for 2 hours at room temperature, then stained with an anti-porcine TLR9 primary antibody (clone CAC-THU-A-TLR9) at 1/300 dilution overnight at 4 °C, followed by rinsing in TBS + 0.025% Triton X-100 for 5 minutes, then staining with a horseradish peroxidase-conjugated secondary antibody for 1 hour at room temperature.

### Statistics

Standard descriptive statistics were used to evaluate all data. Mitochondrial DNA concentrations were compared using a nonparametric one-way Kruskal-Wallis and *post hoc* Dunn’s tests. Continuous variables of mean fluorescence intensity (MFI) for phenotyping and respiratory burst by FACS, inflammatory marker concentrations and transcription, and NE activity were compared against samples from peripheral blood neutrophils using nonparametric Wilcoxon Mann-Whitney U tests, with histology data compared by student’s *t*-test. All data were non-randomized and analyzed using SPSS v.17.0 (SPSS Inc, Chicago, IL, USA).

## Results

### Elevated neutrophil cell and mtDNA levels are present during bladder injury

**Figure 1:**
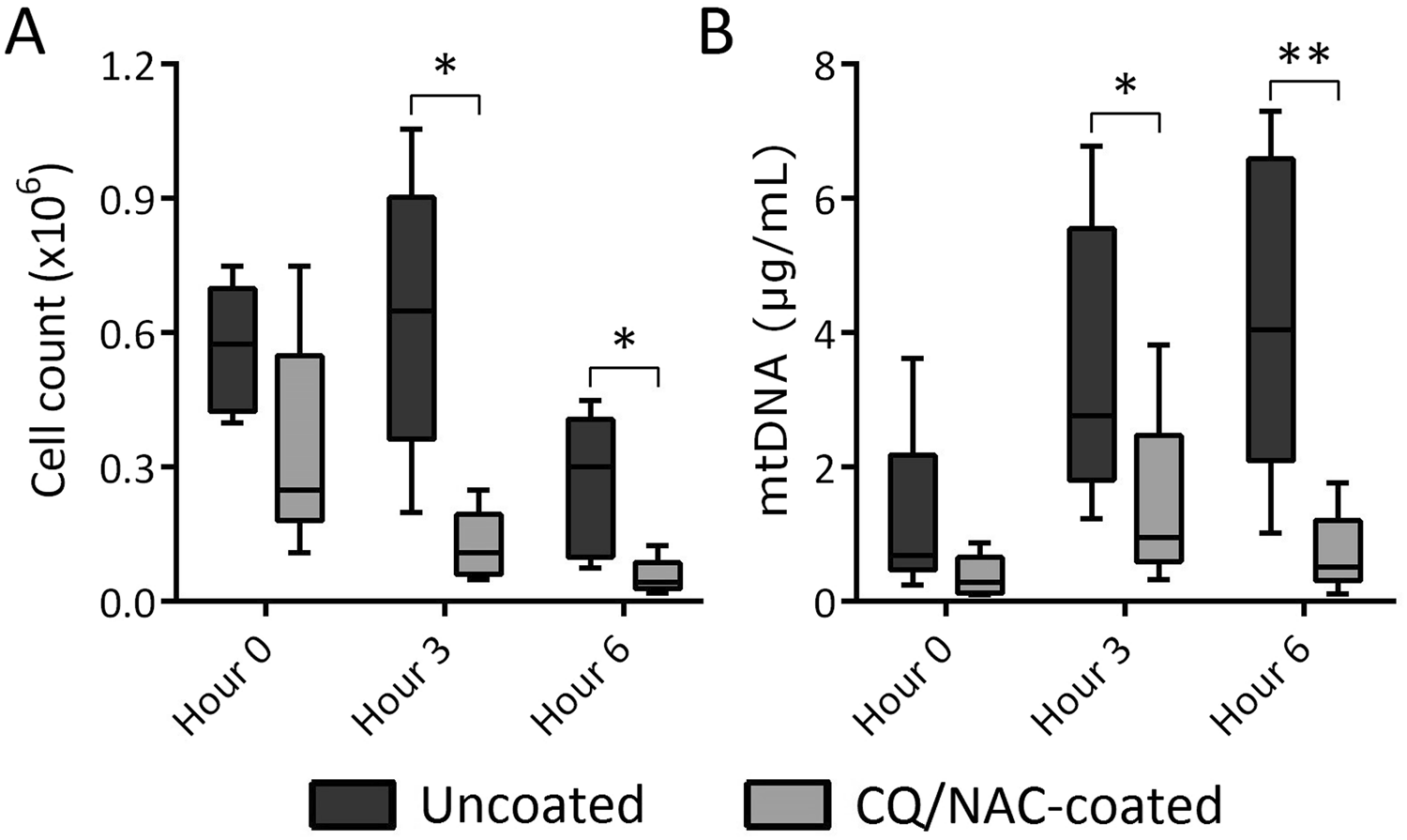
High urine neutrophil numbers and mitochondrial DNA concentrations, but low bacterial DNA, found in uncoated Foley catheter (FC) swine. (**A**) Total live neutrophils were counted by trypan blue exclusion after negative immunomagnetic isolation in both coated (light gray, n = 5) and uncoated (dark gray, n = 5) FC groups. (**B**) Mitochondrial DNA concentrations were determined by real time PCR using swine *Mt-cyb* primer and a standard provided by the manufacturer, *p < 0.05, **p < 0.01.

Extracellular mtDNA has been found in the circulation of patients with sterile injury such as myocardial infarction^43^ and trauma^29^, where it had been implicated in TLR9 activation. It is also known that ligation of TLR9 results in NF-κB activation^44^. Previously, we detected mtDNA during tracheal tissue injury^33^, hence our interest in measuring mtDNA in the urine and its effect on TLR9 levels and NF-κB activity following FC placement. In the current study we documented mtDNA local levels that closely resemble the systemic levels observed in trauma^28^ patients in which systemic inflammation was documented. The mtDNA elevation noticed in the present study appears to follow an upward trend during the 6 hours of the experiment in the uncoated FC group, whereas, a downward trend was noticed in the neutrophil counts after 3 hours for both the coated and uncoated groups. Interestingly, although the overall difference between neutrophils form the coated and uncoated groups remained significant, the decrease in neutrophil counts can be explained in part by a high necrosis rate in the uncoated group, thus partially explaining the persistently elevated mtDNA. It is not possible here to exclude the possibility that mtDNA was release by not only neutrophils but also other cells present in the local milieu, such as macrophages and epithelial cells (Fig. 1A,B). Our findings suggest that various factors are likely involved in bladder inflammation induced by FC, and that mtDNA levels generated by cellular injury had a prominent role.

### Bladder instrumentation induced unique neutrophil phenotype and cell necrosis

**Figure 2:**
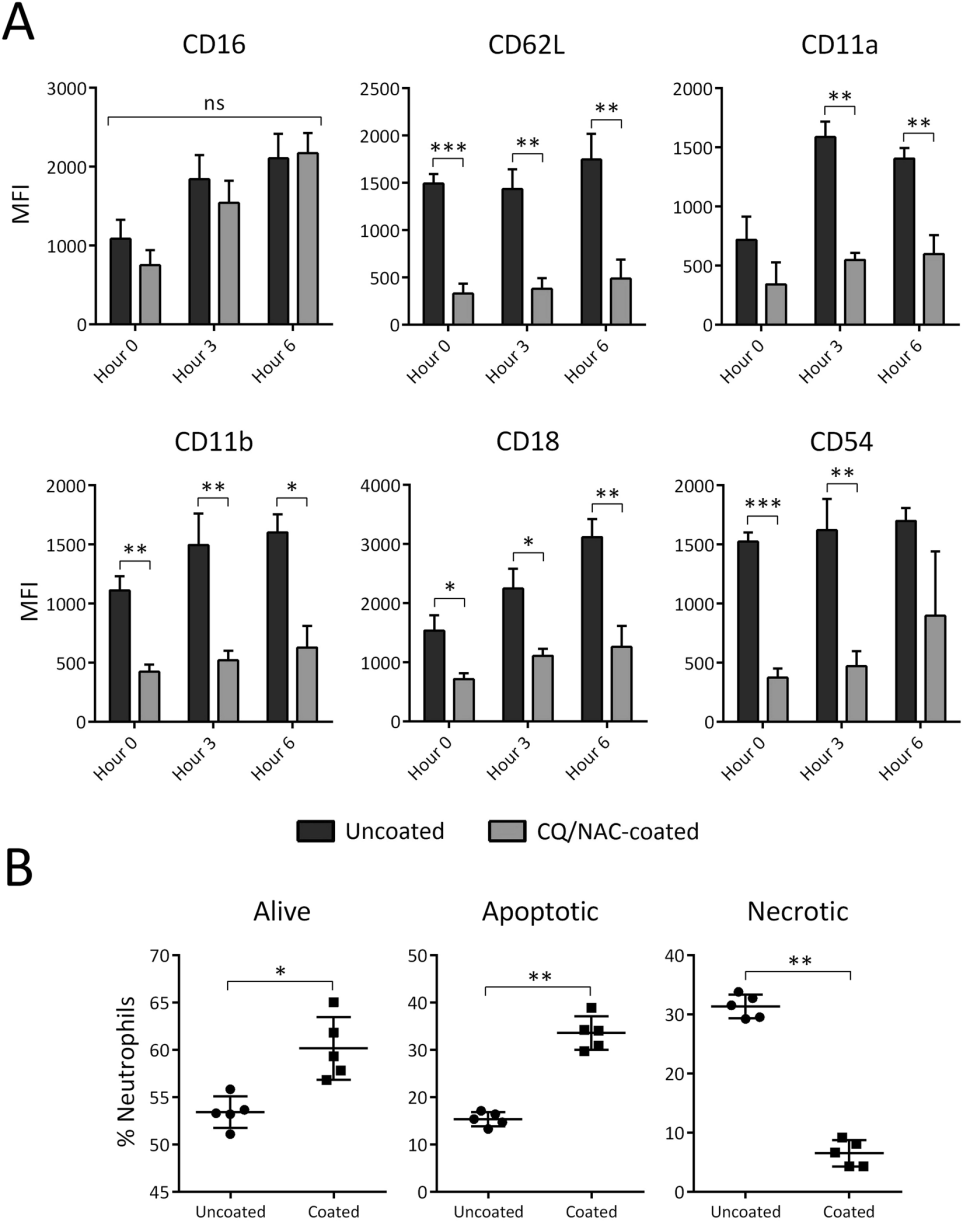
Unique urine neutrophil phenotype correlates with cellular necrosis during Foley catheter (FC)-mediated injury. (**A**) Fluorescence activated cell sorting (FACS) analysis of active urine CD16/CD62L neutrophils indicating different migration marker phenotypes of CD11a, CD11b, CD18, and CD54. (**B**) Alive, apoptotic, and necrotic neutrophil percentages were determined by FACS analysis by Annexin V side scatter and 7-Aminoactinomycin D forward scatter gating for both uncoated and coated FC groups. Data shown represents mean MFI + SD, *p < 0.05, **p < 0.01, ***p < 0.001.

Previously, we have shown that neutrophilia in the luminal space is a common response during human and swine airway mucosal invasion by a foreign body^5,6^, an observation that lead us to investigate if similar cellular activity was present in the swine bladder. Luminal urine neutrophils were identified by FACS using monoclonal antibodies to stained surface markers indicative of neutrophil cells as determined by SCC^hi^ CD62L^lo-hi^ CD16^lo-hi^ and analyzed independently for the coated and uncoated Foley groups. Interestingly, there were distinctive activation patterns for the phenotype that corresponded to uncoated Foley subjects as follows: CD62L^hi^, CD16^lo^, CD54^hi^, CD11b^hi^, and CD18^hi^, whereas a less activated phenotype CD62L^lo^, CD16^lo^, CD54^lo^, CD11b^lo^, and CD18^lo^ predominated in the coated group. The CQ/NAC coated group for instance displayed a lower MFI for CD54 (ICAM-1), a protein expressed in neutrophil cells after trans-epithelial migration, whereas the ICAM-1 that corresponded to the uncoated group was significantly higher. Furthermore, as a result of the neutrophil activation observed in our study, we documented elevation of CD11a and CD11b adhesion molecules known to bind to CD54^45^, and found to be decrease during exposure to CQ/NAC (Fig. 2A).

Next, we examined neutrophil viability using FACS analysis of the two neutrophil phenotypes found by using 7AAD to determine cell necrosis, and the calcium-dependent protein Annexin-V markers with the purpose of determining live cells undergoing natural apoptosis. Clearly, there were a greater proportion of necrotic cells (32%) in the uncoated FC group, whereas apoptotic (33%) and live cells (60%) were highly represented in the coated FC group (Fig. 2B). Our findings support the benefit of CQ/NAC in ameliorating neutrophil activation by a better preservation of live cells that will undergo a natural cell death by apoptosis and thus facilitating efferocytosis by macrophages, whereas lower percentage of live cells in combination with high concentration of necrotic cells noticed in the uncoated group likely represents a meaningful source of DAMPs (mtDNA).

### Luminal urine neutrophils express TLR9/NF-κB during activation

**Figure 3:**
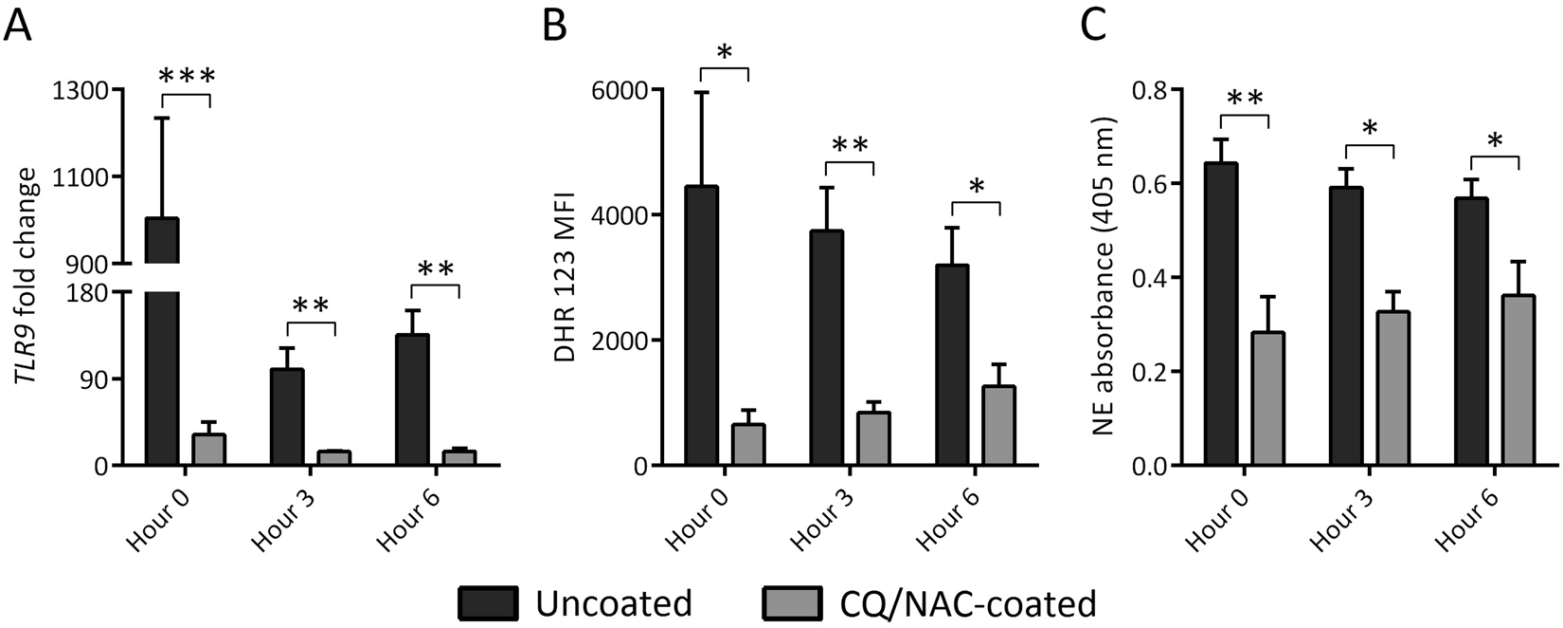
Foley catheters (FCs) promote reactive oxygen species (ROS) and neutrophil elastase (NE) activity in a TLR9 dependent manner. (**A**) Fold change in TLR9 transcription in urine neutrophils from uncoated (dark gray, n = 5) and coated (light gray, n = 5) FC groups as determined by real time PCR against peripheral blood neutrophils. (**B**) ROS production by these neutrophils in both FC groups at each time point by dihydrorhodamine (DHR) 123 staining fluorescence activated cell sorting (FACS) analysis. (**C**) Urine neutrophils from both groups were assessed for NE activity after 1-hour incubation with NE-specific substrate by spectrophotometry. Data shown as mean + SD, *p < 0.05, **p < 0.01, ***p < 0.001.

TLR9 is a pattern recognition receptor capable of recognizing hypomethylated motifs found in mitochondria DNA and DNA from pathogens^29,46,47^. TLR9 protein levels were measured in luminal neutrophils isolated from urine specimens collected at several time intervals. As we hypothesized, increased expression of TLR9 (Fig. 3A) correlated with an increase in urine mtDNA concentration, reactive oxygen species (ROS) production (Fig. 3B), and elastase activity (Fig. 3C) by urine neutrophils. We demonstrated that activation of TLR9 by CpG DNA was sufficient to induce pro-inflammatory cytokine transcription and secretion mediated by NF-κB gene expression during *in vivo* and *in vitro* experiments. Interestingly, in the uncoated group as expected, a significant increase of TLR9 activity was detected early in the experiments, whereas unexpected lower levels were observed during the subsequent time points. The observed trends suggest that presence of a naturally occurring TLR9 antagonist is keeping the levels lower, albeit higher than the levels observed in the CQ/NAC treated group. Also, the detection of the anti-inflammatory cytokine IL-10 at very low levels in the uncoated group raises the possibility that IL-10 presence even at low levels may be sufficient to ameliorate the inflammatory response to a FC under natural conditions^48,49^. However, we must consider the impact of an inhibitory oligonucleotide^50^, yet to be identified, on TLR9 activity as illustrated in the HEK-Blue™ hTLR9 reporter cell line. A noticeable decrease in TLR9 levels was documented in our study with CQ/NAC alone and in combination. Our study was designed to determine if mtDNA impacted TLR9 activation in neutrophil cells and we did not attempt to determine the mechanisms involved in extracellular mtDNA uptake and internalization that resulted in TLR9 activation. With the use of CQ a known inhibitor if endosomal acidification we demonstrated inhibition of TLR9 transcription (Fig. 3A) *in vivo* using a coated FC in direct contact with the urethra and bladder tissue and was confirmed *in vitro*. A controlled process of neutrophil apoptosis is essential for prevention of inflammation, however delayed apoptosis as induced by TLR9 or trigger during trafficking may have a negative impact on tissue homeostasis^51,52^.

### Coated Foley catheters ameliorate bladder inflammation and modify TLR9/NF-κB activity

**Figure 4:**
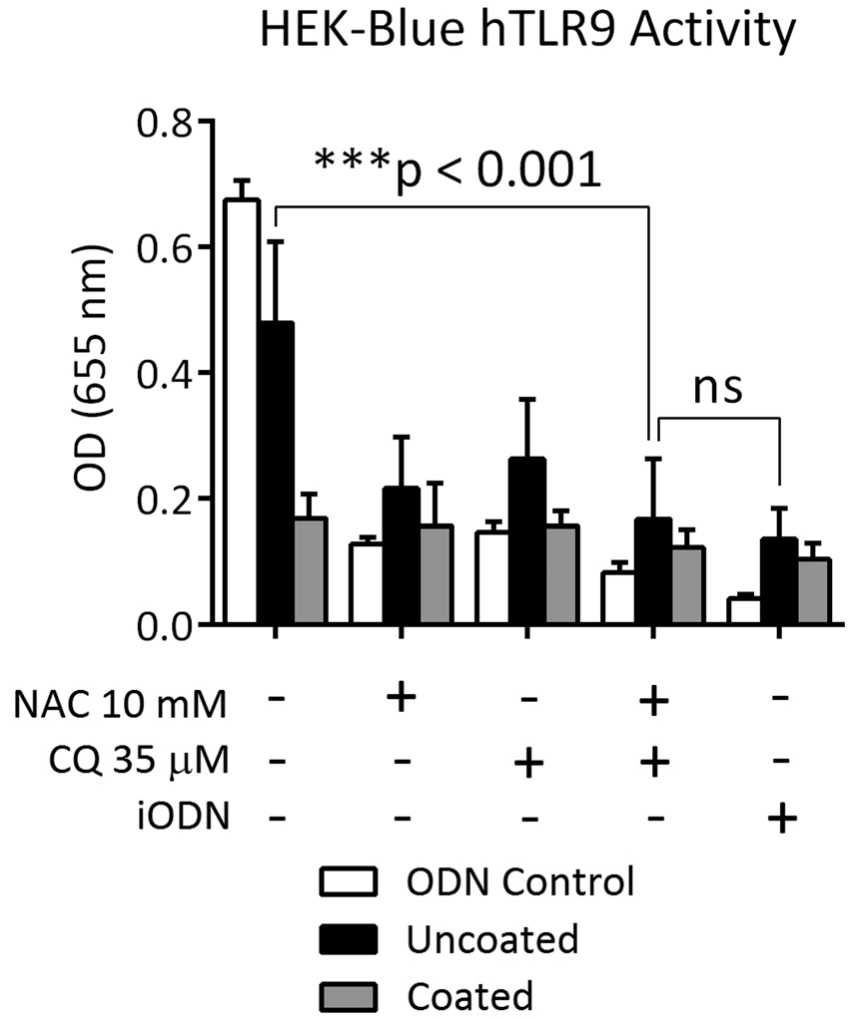
Urine mitochondrial DNA induces TLR9 activity. Urine specimens obtained from uncoated or CQ/NAC coated FC were co-incubated with HEK-Blue™ hTLR9 reporter cell line or pre-incubated with graded concentrations of NAC or CQ alone or TLR9 iODN. After 6 hours supernatants were analyzed for activity by spectroscopy of the target transgene NF-κB-induced secreted embryonic alkaline phosphatase absorbance at 655 nm. Data is representative of triplicate experiments where mean activity + SD was calculated from 5 coated and 5 uncoated FC subjects. HEK – human embryonic kidney; iODN – inhibitory oligodeoxynucleotide; NAC – N-Acetylcysteine; CQ – chloroquine. ***p < 0.001.

Here, we documented mtDNA in the urine at levels that resemble concentrations found in other studies^29^ in which inflammation was mediated by TLR9 activation, followed by NF- κB mediated cytokine production^53,54^. To determine if the urine mtDNA could be driving TLR9 and subsequent NF-κB activation directly, we assessed urine samples for activation of the TLR9/NF-κB signaling pathway in a HEK-Blue™ hTLR9 reporter cell line and demonstrated that uncoated FC urine samples showed a significant increased of NF-κB activation as measured by SEAP production (Fig. 4), whereas urine from CQ/NAC coated FC urine samples showed significantly less activation as measured by SEAP production. Similarly, co-incubation of urine samples with NAC, CQ, CQ/NAC, and the inhibitory oligodeoxynucleotide (iODN) all caused a noticeable decrease in SEAP production.

**Figure 5:**
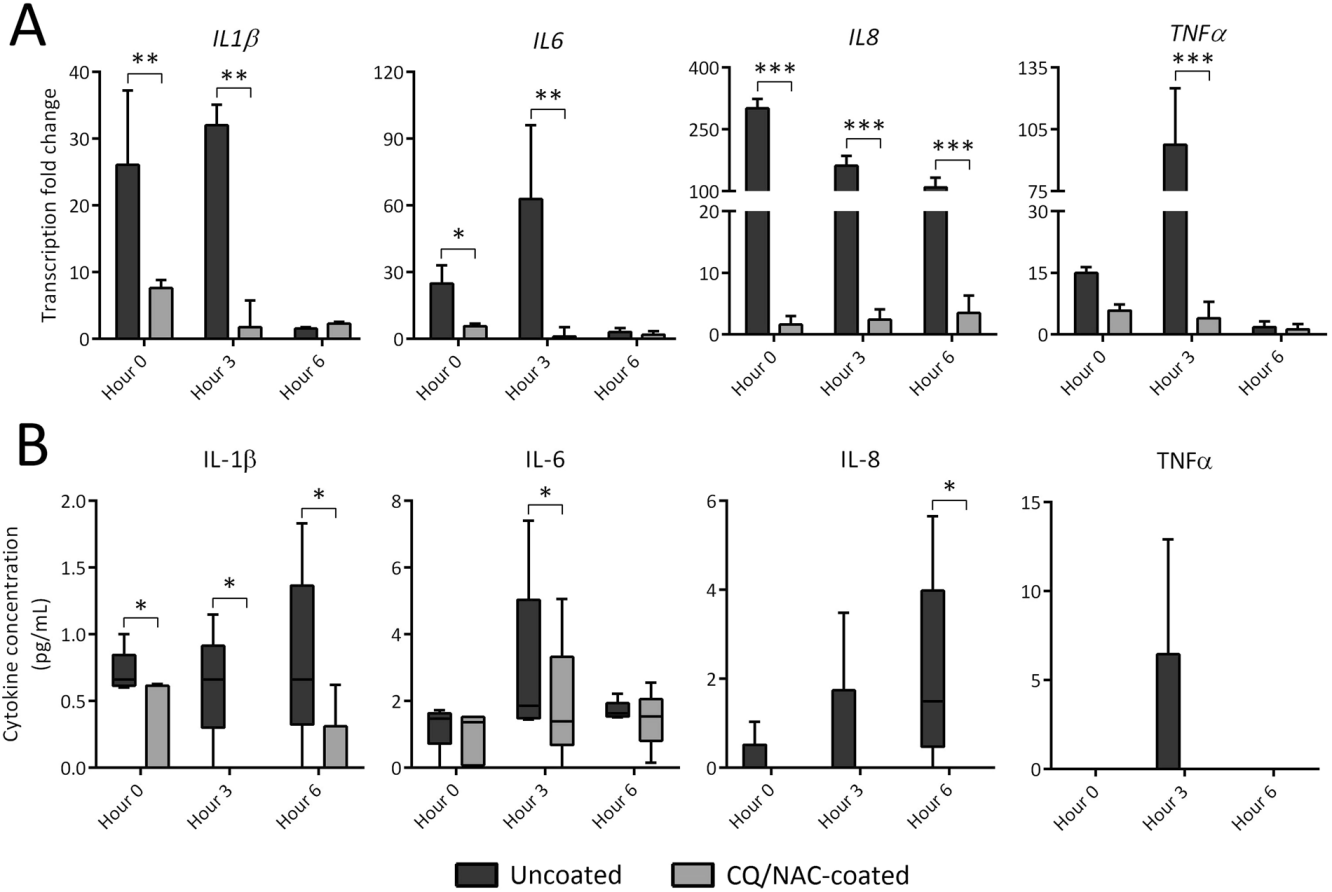
Urinary cytokine concentrations and neutrophil cytokine transcription is mediated by chloroquine/N-Acetylcysteine (CQ/NAC) coating. (**A**) Fold change in transcription of these pro-inflammatory cytokines in urine neutrophils from both FC groups determined real-time qPCR measured against peripheral blood neutrophils, with data shown as mean fold change + SD. (**B**) Concentrations of pro-inflammatory cytokines found in approximately 20 mL urine from both uncoated (dark gray, n = 5) and coated (light gray, n = 5) Foley catheter (FC) groups determined by enzyme linked immunosorbent assay (ELISA), with data shown as box-and-whisker plots with standard descriptive statistics. *p < 0.05, **p < 0.01, ***p < 0.001.

**Figure 6:**
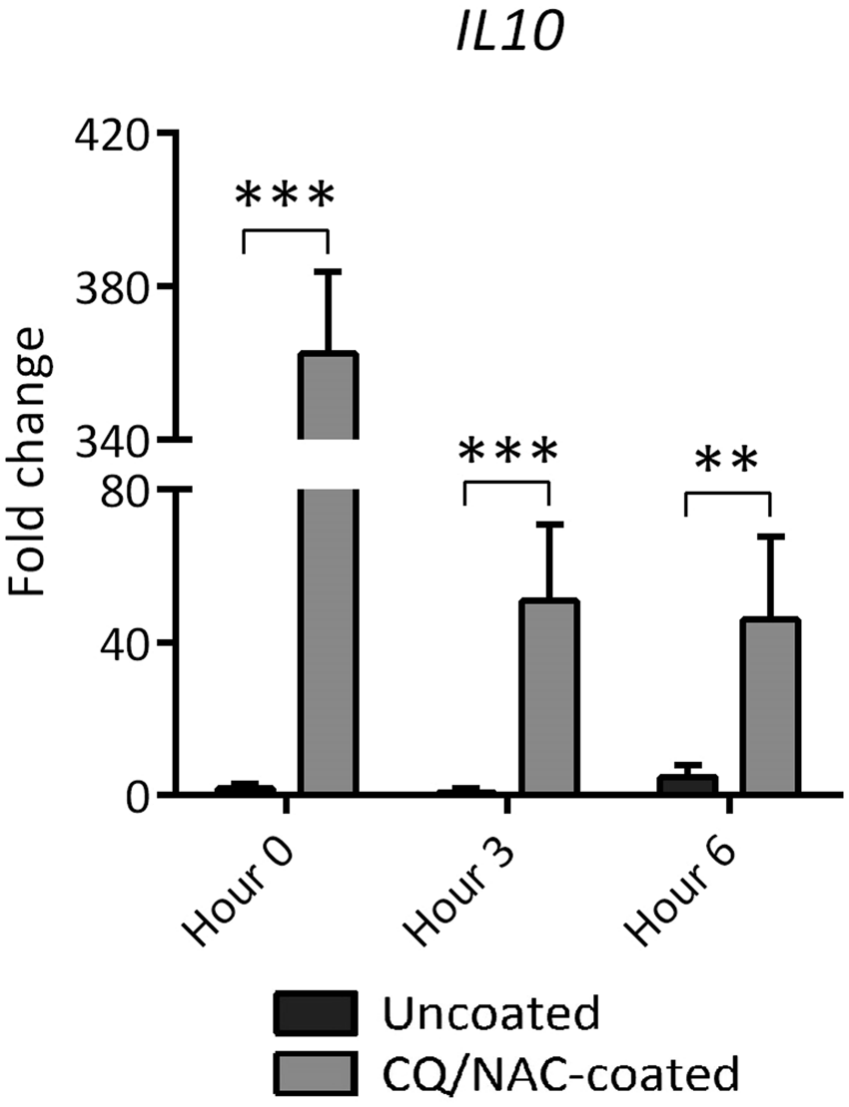
Coating Foley catheters (FCs) with chloroquine and N-Acetylcysteine promotes transcription of the anti-inflammatory cytokine IL-10. Messenger RNA from urine neutrophils of coated and uncoated FC groups was isolated and assessed for transcription of the anti-inflammatory cytokine IL-10 by real time PCR measured against peripheral blood neutrophil mRNA. Data shown represents results of at least 10 experiments, 5 per group, with mean fold change + SD. **p < 0.01, ***p < 0.001.

Next, we measured production of pro-inflammatory cytokines IL-1β, IL-6, IL-8 and TNF-α in luminal bladder neutrophils by qPCR (Fig. 5A) and Luminex (Fig. 5B) because these genes/proteins are believed to increase in response to mtDNA activation of TLR9/ NF-κB. The neutrophil gene expression results from the uncoated FC group show significantly higher levels than the gene expression levels from neutrophils isolated from urine from the CQ/NAC coated FC group (Fig. 5A). These results support our belief that mtDNA released by necrosis in the uncoated FC group activates TLR9 to induce NF-κB gene transcription of each of the cytokines measured. Likewise, the pro-inflammatory cytokine levels in the urine from the uncoated FC group were significantly higher than the urine cytokine levels from the CQ/NAC coated FC group (Fig. 5B). Interestingly, little or no measureable gene transcription of these cytokines was detected in the luminal neutrophils from the CQ/NAC coated FC group. Interestingly, transcription of the anti-inflammatory cytokine IL-10 correlated with a decrease in the pro-inflammatory cytokines suggesting an innate effort by the local tissues to prevent further damage^49^ (Fig. 6). IL-10 had no apparent effect on TLR9 gene expression (Fig. 3A) suggesting that different pathways are engaged.

### Bladder histology and immunohistochemistry shows diffuse injury and TLR9 expression in the uncoated FC group

**Figure 7:**
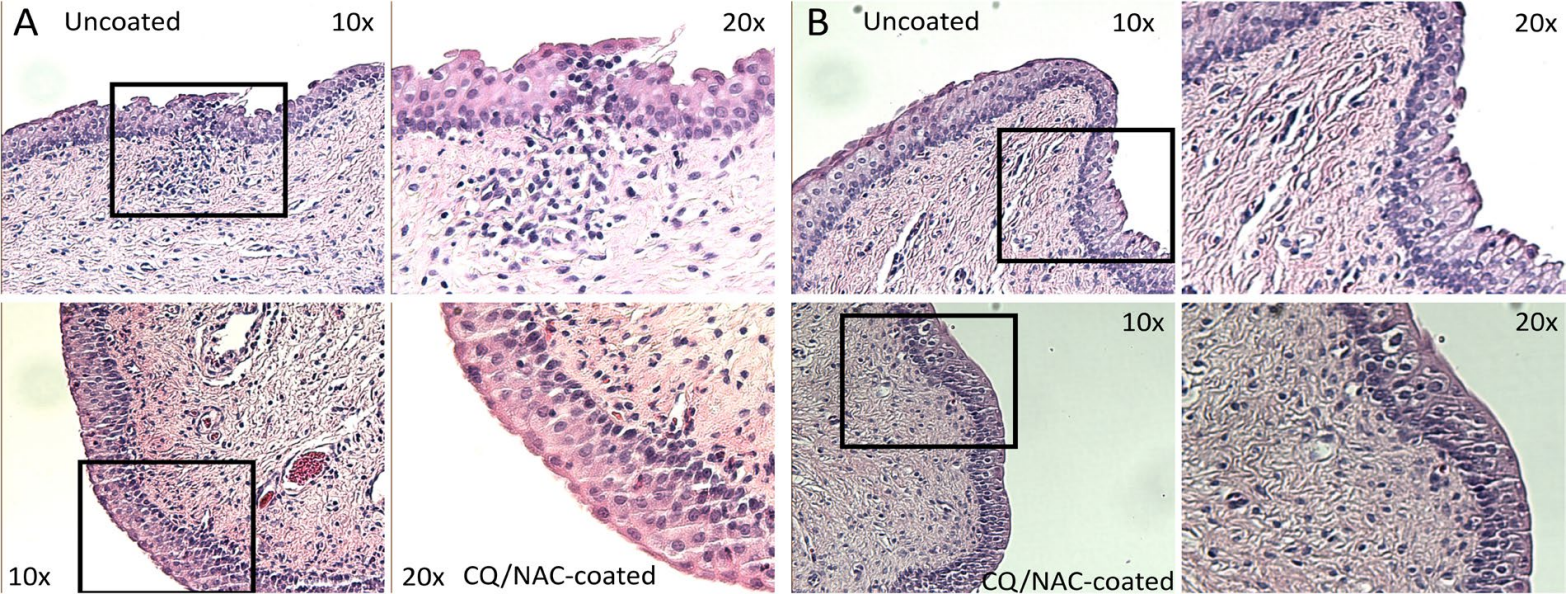
Urethra and bladder neck histology from uncoated and coated Foley catheters (FCs). After sample preparation, paraffin-embedded blocks were cut and stained with H&E. Tissue samples at point of contact with uncoated (top) and coated (bottom) FCs in the urethra (**A**), and bladder neck (**B**) at 100× magnification with boxes and 200× magnification highlighting distinct morphological and immune cell infiltration differences.

**Figure 8:**
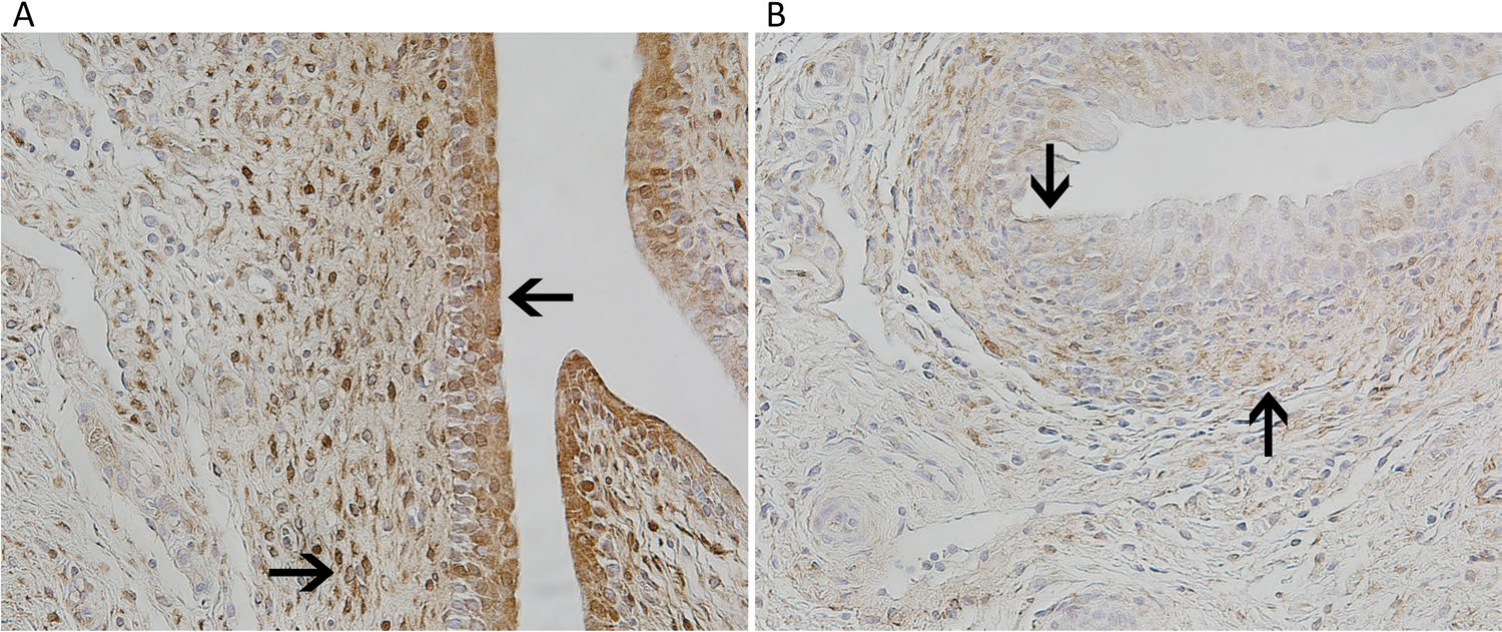
Urethra immunohistochemistry of TLR9 staining from uncoated and coated Foley catheters (FCs). After preparation, paraffin-embedded tissues were deparaffinized and prepared for IHC staining for porcine TLR9 with horseradish peroxidase secondary staining. Tissue samples at point of contact in the urethra for uncoated (**A**) and CQ/NAC-coated (**B**) FCs at 200× magnification. Arrows point to notable areas on the epithelial surface and subepithelial layer to show differences in TLR9 expression.

After sample preparation and staining, a veterinary pathologist blinded to the samples examined urethra (Fig. 7A) and bladder neck tissue samples (Fig. 7B) from both the coated and uncoated FC groups. Comparative histology between CQ/NAC coated and uncoated urethra/bladder wall tissues showed a higher score (1.25) in the uncoated tissues indicating mild congestion, some edema and minimal hemorrhage, whereas the tissues exposed to a coated Foley catheter had a much lower score (0.33) indicating very good preservation of tissues (Table 1). Similar numbers of neutrophil cells in the coated and uncoated tissues likely indicate post-migration levels in the uncoated specimen in comparison with the resident neutrophil cell levels likely present in coated tissues. These results aligned with the high luminal neutrophil migration detected in the uncoated group and corresponded to disruption of epithelial tissues. Likewise, immunohistochemical staining shows that TLR9 expression is markedly higher in the uncoated FC group (Fig. 8A) compared to the coated group (Fig. 8B).

**Table 1:** Histology of urethra and bladder tissues at hour 6 scored for pathology and infiltration of inflammatory cells, shown as mean (SD). *p < 0.05, **p < 0.01 by student’s *t*-test.

**Variable**	**Uncoated**	**Coated**	***p***
Criterion:			
Fraction of basement membrane covered by epithelium	1.25 (0.25)	0.33 (0.33)	0.01**
Bladder wall pathology	1.83 (0.23)	1.5 (0.16)	0.037*
Inflammation – Neutrophils	1 (0.17)	0.92 (0.12)	0.407
Inflammation – Mononuclear cells	2.33 (0.08)	2.25 (0.10)	0.202
Average Total Score	6.42 (0.55)	5 (0.87)	0.015*

## Discussion

Instrumentation of the bladder mucosa may induce cystitis, bladder spasms, pain, and urinary infection mediated by activation of the innate immune system, specifically neutrophils^1,2,12^. Our previous work in mucosal inflammation^5,6,33^ suggests that multiple factors may be involved in innate immune response to the presence of a foreign body in contact with mucosal tissues, in particular inflammation in the absence of infection, thus we focused our attention on sterile triggers of inflammation. Mucosal tissues are exposed to a variety of bacterial and host-derived signals that require a well-organized neutrophilic response to preserve tissue homeostasis. However, uncontrolled neutrophilia in the bladder lumen implies disruption of the epithelial layer, and suggests trafficking of neutrophils towards a chemotactic gradient^36,37^. The combination of tissue damage and neutrophil migration across epithelial layers is generally attributed to degranulation^54^ of neutrophils and release of proteinases such as neutrophil elastase^55^, matrix metalloproteinases, and cathepsin G. These proteinases promote tissue invasion or protein degradation as determined by post-translational modification of proteins. As a result of migration across epithelial tissues, neutrophils may have enhanced activation and degranulation^56,57^, thus increasing release of myeloperoxidase and neutrophil elastase leading to further cellular injury and necrosis. Necrotic cell death will release intracellular damage associated molecular patterns (DAMPs), in particular mitochondrial DNA (mtDNA). MtDNA is a TLR9 ligand that is recognized and internalized by neutrophils using mechanisms that are not completely understood. Activation of TLR9^46,47^ promotes further inflammation by downstream engagement of NF-κB resulting in production of pro-inflammatory cytokines. Disruption of the inflammatory process in mouse models of lung tissue necrosis and injury has been accomplished with *tlr9* gene deletion^34^. Similarly, we studied the administration of chloroquine (CQ), an antimalarial drug known to interfere with endosomal activation of TLR9^38,39^, and N-Acetylcysteine (NAC), an antioxidant and inhibitor of NF-κB^40,41^ to determine their potential local therapeutic benefits when used to coat Foley catheters.

We examined neutrophil concentrations and viability in urine specimens and documented a significant elevation of neutrophils and necrosis in the uncoated group, thus providing a likely source of DAMPs, in particular mtDNA. However, it is possible that neutrophils are not the only source of DAMPs as epithelial cells may also play a role in bladder inflammation. This study demonstrates that placement of a Foley catheter results in bladder tissue injury within three hours of exposure, resulting in neutrophil migration and activation. Notably, neutrophil migration indicates disruption of the epithelial layer of the bladder and is generally associated with cellular degranulation and proteinase (e.g., neutrophil elastase) activity. As the neutrophils migrate through the epithelium, local surface proteins become activated as noticed here in which CD54 (ICAM-1), a protein expressed in the epithelium, likely imparted activity to neutrophil ICAM-1 and CD18. Evidence of neutrophil activation has also been reported in ischemic injury as determined by CD11b and CD54 expression^58,59^. For example, CD54 further amplifies inflammation by combining with other neutrophils and macrophages expressing CD11b^60^.

As a result of sustained trauma to the bladder, further accumulation of neutrophils at different stages of activation takes place. For example, we documented the presence in the bladder lumen of an activated neutrophil phenotype characterized by high surface protein membrane expression of CD11a, CD11b, CD18, CD54, and CD62L, which is consistent with activation and migration of local neutrophils. We can speculate that the phenotype we regarded as not activated may have indeed been the first active neutrophil group and now are depleted of any obvious immune activity.

Progressive activation and degranulation of neutrophils leads to amplification of local inflammation by releasing sustained levels of reactive oxygen species (ROS), neutrophil elastase, and pro-inflammatory cytokines. For instance, extracellular release of ROS and elastase indicates a process of degranulation since neutrophils contain azurophilic granules rich in these pro-inflammatory factors. Furthermore, elastase may also participate in neutrophil extracellular trap (NET) formation where it serves several purposes including chromatin decondensation and acts in synergy with myeloperoxidase during immune responses. ROS are present during normal cellular activity, however persistent elevation as the one observed in our current study may have a negative impact on tissue homeostasis due to their ability to damage macromolecules (i.e., DNA, RNA, lipids, carbohydrates, and proteins) in neighboring cells^61–63^, thus inducing bladder tissue injury. ROS and elastase levels were significantly decreased in the CQ/NAC-coated FC group.

As a result of cellular injury, host sterile intracellular molecules such as DAMPs^20–22^ such as mtDNA are released to the extracellular compartment. We documented progressive elevation of mtDNA in urine specimens containing high numbers of neutrophils, and elevated ROS activity and elastase concentrations, indicating that these cells are likely releasing mtDNA during the process of necrosis. Interestingly, others have documented the presence of high systemic mtDNA in sterile injury that resulted in organ dysfunction^22,27,29,32,33,43^, whereas in our study we show similar mtDNA levels but localized in the bladder. More recent work has demonstrated that oxidized mtDNA released during eukaryotic cell injury induces neutrophil expression of TLR9, thus mediating neutrophil activation^27^. We also confirmed that the mtDNA concentration in the urine of uncoated Foley catheters was sufficient enough to upregulate TLR9 activity and induce NF-κB activation, as assessed in the HEK-Blue™ hTLR9 cell reporter line. It is also conceivable that mtDNA release is not exclusively originating from neutrophils, but also the epithelium.

Neutrophils utilize Toll-like receptors (TLRs) to recognize pathogen (PAMPs) and damage (DAMPs) associated molecular patterns^18,19,23^. PAMPs activate various TLRs (e.g., TLR4, TLR5, and TLR11) in the urinary system^18^, where they induce pro-inflammatory gene expression following infection. TLRs, and in particular TLR9, can be activated by evolutionarily conserved molecular patterns such as hypomethylated CpG motifs present in both bacterial DNA (PAMPs) and oxidized endogenous mtDNA (DAMPs)^25–27^. Similarly, TLR4 activation has been demonstrated in response to bacterial lipopolysaccharides (LPS) leading to NF-κB activation and production of the pro-inflammatory cytokines TNF-α, IL-6, and IL-8^18,23^. Impaired activity of TLR4 will result in urinary infections^23^. Interestingly, there are reports of crosstalk between TLR4 and TLR9 in macrophages^24^ resulting in amplification of the inflammatory response observed in mouse models. Therefore, we are unable to completely exclude some form of cooperation between these two TLRs, despite targeted stimulation from different sources^24,52^. Our urine analysis of bacterial *16S rRNA* DNA showed nearly undetectable levels of bacterial contamination in both coated and uncoated urine specimens (data not shown), consistent with our observation that the leading inflammatory source of bladder injury in this case is mtDNA. TLR9 activation relies on endosomal compartment modification to perform its function as a sensor for endogenous DAMPs such as mtDNA, which induce pro-inflammatory gene expression in neutrophils^64^. Hence our interests in modifying the endosomal pH with chloroquine. Our findings highlight the prominent role of mtDNA in acute bladder injury mediating activation of the TLR9/NF-κB pathway.

Innate immune cells naturally die by apoptosis, which serves multiple purposes including the resolution of inflammation. However, various factors may delay apoptosis, thus exposing local tissues to uncontrolled neutrophil migration and activation^54^. Also, TLR9 has been implicated in delaying neutrophil apoptosis by increasing transcription of anti-apoptotic genes following exposure to bacterial DNA^51,52^. In this current study, during exposure to meaningful concentrations of mtDNA the percentage of necrotic cells in urine samples was high while the percentage of apoptotic cells was low suggesting that during the early stages of bladder injury apoptotic delays are not likely a predominant factor but rather necrosis appears to lead the initial injury. Opposite effects were noticed in the CQ/NAC-coated group.

Neutrophils are essential for a proper innate immune response to both infectious and non-infectious antigens^11,12^ and part of the acute response includes activation and migration^55–57^ through epithelial layers following chemotactic factors^36,37^. Here we demonstrated that mtDNA originating from necrotic cells promotes activation of the TLR9/NF-κB pathway resulting in chemokine and cytokine transcription and secretion. Although studies have shown cytokine release following urinary infections^14,17^, the impact of sterile bladder injury induced by DAMPs such as mtDNA is less well defined. To this end, we observed higher transcription and secretion of the pro-inflammatory cytokines IL-1β, IL-6, and TNF-α during activation of the TLR9/NF-κB pathway at the time of exposure to uncoated Foley catheters. Among the neutrophil functions known to occur during an injury is the capacity to migrate towards a chemotactic signal released during cellular stress^54,56^, and in that regard, we documented the presence of the chemokine IL-8 in the urine samples that correlated with high neutrophil counts and corresponded to the uncoated FC group. This study showed that *IL8* gene transcription negatively correlated with *IL10* transcription in the coated FC group, a result that may have an effect on chemokine activity as IL-8 binds to CXCR1 and CXCR2, thus impacting degranulation and amplification of the inflammatory response. IL-8 levels were significantly lessened by exposure to coated Foley catheters. Furthermore, IL-1β^65^ has been implicated in inflammasome formation via caspase-1 activation as a result of the cytosolic release of mtDNA, suggesting that this mechanism may have a role in the pathophysiology of bladder inflammation. Several of the cytokines analyzed in the current study (i.e., IL-1β and TNF-α) have been implicated in pain^66,67^, particularly during simultaneous elevation of neutrophil elastase. Interestingly, transcription of the anti-inflammatory gene *IL10* may not only participate in reducing inflammation but also in regeneration of epithelial tissues^26,27,33^ as seen during mast cell activation, and thus its role in the CQ/NAC-coated group may also play a role in protecting the epithelium from damage.

Finally, confirmation of the beneficial impact of CQ/NAC was observed by histological evaluation indicating less tissue damage, better epithelial integrity and lower injury scores. Interestingly, the tissue cellularity appears to indicate that neutrophil migration into the lumen has occurred and cell counts in the tissues are at a transitional stage in preparation for a new influx of cells. Similarly, tissues from the CQ/NAC-coated FC group show marked reduction in TLR9 expression in the epithelium by immunohistochemical staining compared to the tissues from the uncoated FC group, indicating a protection from TLR9-mediated inflammation and damage. Thus, our study confirms the benefits of administration of chloroquine and N-acetylcysteine likely due to interference with endosomal activation of TLR9 and inhibition of NF-κB, respectively.

Some limitations of our study will be addressed in future studies and will include extending the study period to more than six hours to evaluate the full impact of epithelial damage in promotion of bacterial infiltration and seeding over a clinically relevant period of time. Further analysis of TLR9 interactions with other TLRs is also necessary, as TLR4/TLR9/CD14 interactions have not been analyzed in the context of bladder inflammation mediated by sterile factors.

In summary, the findings of this study provide supporting evidence to explain the initial pathological events leading to bladder tissues damage during exposure to a Foley catheter and illustrate the importance of the proper activation of the innate immune system to control and prevent epithelial damage. Most studies focus on the impact of bacterial inflammation in tissue damage, neglecting early sterile inflammatory events as the ones presented in this study. A better understanding of the factors involved in sterile injury and the impact of early intervention to prevent cellular activation and tissue damage may have beneficial effects on clinical symptoms and outcomes regarding pain and recurrent infections.

We demonstrated that cellular necrosis induced by a Foley catheter has a prominent role in releasing mtDNA, which in turn leads to activation of the TLR9/NF-κB pathway and induces pro-inflammatory marker, elastase, and ROS production and release, thus resulting in tissue damage. Our findings indicate that upstream interventions to prevent TLR9 activation at the endosome in combination with amelioration of NF-κB activity using CQ/NAC-coated FCs results in a short-term decrease in bladder tissue damage, thus preventing uncontrolled activation of neutrophils. A better understanding of the signaling pathways involved in bladder injury is necessary to devise new therapies, and our initial results indicate that amelioration of injury and bladder tissue preservation are a feasible goal to improve health.

## Electronic supplementary material

Supplemental Table 1
