# Supplementary material for: Mitochondrial DNA induces Foley catheter related bladder inflammation via Toll-like receptor 9 activation
Source: Sci Rep. 2018 Apr 23;8:6377. doi: 10.1038/s41598-018-24818-w (PMC5913242; doi:10.1038/s41598-018-24818-w)
Supplement: Supplementary file 1 — Supplemental Table 1 [file 41598_2018_24818_MOESM1_ESM.pdf]

Mitochondrial DNA induces Foley catheter related bladder inflammation via Toll-like receptor 9 activation

Carlos A. Puyo[1,*], Alexander Earhart[1], Nicholas Staten[1], Yuan Huang[1], Alana Desai[2], Henry Lai[2], Ramakrishna Venkatesh[2]

[1]Department of Anesthesiology and Critical Care, Washington University School of Medicine in St. Louis, Missouri USA. [2]Department of Surgery, Washington University School of Medicine in St. Louis, Missouri USA

*Correspondence and requests for reprints can be addressed to:

Carlos A. Puyo
Assistant Professor
Department of Anesthesiology and Critical Care
Washington University School of Medicine in St. Louis
Telephone: 1-314-747-0259
Fax: 1-314-362-8571
E-mail: puyoc@wustl.edu

Running title: Neutrophil activation from Foley catheterization

Keywords: neutrophils, toll-like receptor 9, mitochondrial DNA, cystitis

Criterion	Fraction of basement membrane covered by epithelium	Bladder wall pathology	Neutrophils*	Mononuclear cells*
Score:				
0	>80%	No significant change	0 HPF	0 HPF
1	65-80%	Minimal to mild congestion, +/- edema and minimal to mild hemorrhage	Rare; 1-5 HPF	Rare; 1-5 HPF
2	50-65%	Moderate congestion, +/- edema and hemorrhage	6-20 HPF	6-20 HPF
3	25-50%	Moderate congestion, +/- edema and hemorrhage, and extensive epithelial necrosis	Heavy (moderate)	Heavy (moderate)
4	<25%	Congestion/hemorrhage/edema; extensive epithelial necrosis and necrosis of subepithelial tissues	Massive (marked)	Massive (marked)

Supplemental Table 1. Bladder pathology scoring scheme. *Assessment of inflammation performed at 10 sites at 400x total magnification, with scores averaged to the nearest 0.5; HPF = hits per field (site).
